# Correlates of poor mental health in early pregnancy in obese European women

**DOI:** 10.1186/s12884-017-1595-y

**Published:** 2017-12-04

**Authors:** Matteo C. Sattler, Judith G. M. Jelsma, Annick Bogaerts, David Simmons, Gernot Desoye, Rosa Corcoy, Juan M. Adelantado, Alexandra Kautzky-Willer, Jürgen Harreiter, Frans A. van Assche, Roland Devlieger, Goele Jans, Sander Galjaard, David Hill, Peter Damm, Elisabeth R. Mathiesen, Ewa Wender-Ozegowska, Agnieszka Zawiejska, Kinga Blumska, Annunziata Lapolla, Maria G. Dalfrà, Alessandra Bertolotto, Fidelma Dunne, Dorte M. Jensen, Lise Lotte T. Andersen, Frank J. Snoek, Mireille N. M. van Poppel

**Affiliations:** 1 Institute of Sport Science, University of Graz, Mozartgasse 14, 8010, Graz, Austria; 2 Department of Public and Occupational Health, Amsterdam Public Health Research Institute, VU University Medical Centre, Amsterdam, the Netherlands; 3 Department of Development and Regeneration KULeuven, University of Leuven, Leuven, Belgium and Faculty of Medicine and Health Sciences, Centre for Research and Innovation in Care (CRIC), University of Antwerp, Belgium and Faculty of Health and Social Work, research unit Healthy Living, UC Leuven-Limburg, Leuven, Belgium; 4 Institute of Metabolic Science, Addenbrookes Hospital, Cambridge, England and Macarthur Clinical School, Western Sydney University, Sydney, Australia; 5 Department of Obstetrics and Gynecology, Medizinische Universität Graz, Graz, Austria; 6 Institut de Recerca de L’Hospital de la Santa Creu i Sant Pau, Barcelona, Spain; 7 CIBER Bioengineering, Biomaterials and Nanotechnology, Instituto de Salud Carlos III, Zaragaza, Spain; 8 Division of Endocrinology and Metabolism, Department of Medicine III, Medical University of Vienna, Vienna, Austria; 9 KU Leuven Department of Development and Regeneration: Pregnancy, Fetus and Neonate, Gynaecology and Obstetrics, University Hospitals, Leuven, Belgium; 10 Department of Obstetrics and Gynaecology Division of Obstetrics and Prenatal Medicine, Erasmus Medical Centre, Rotterdam, The Netherlands; 11 Recherche en Santé Lawson SA, Bronschhofen, Switzerland; 12 Center for Pregnant Women with Diabetes, Departments of Endocrinology and Obstetrics, Rigshospitalet, University of Copenhagen, Copenhagen, Denmark; 13 Division of Reproduction, Poznan University of Medical Sciences, Poznan, Poland; 14 Universita Degli Studi di Padova, Padova, Italy; 15 Università di Pisa, Pisa, Italy; 16 National University of Ireland, Galway, Ireland; 17 Odense University Hospital, Odense, Denmark; 18 Department of Medical Psychology, VU University Medical Centre, Amsterdam, the Netherlands; 19 Department of Medical Psychology, Academic Medical Centre, Amsterdam, the Netherlands

**Keywords:** Mental health, Depression, Pregnancy, Obesity

## Abstract

**Background:**

Depression during pregnancy is associated with higher maternal morbidity and mortality, and subsequent possible adverse effects on the cognitive, emotional and behavioral development of the child. The aim of the study was to identify maternal characteristics associated with poor mental health, in a group of overweight/obese pregnant women in nine European countries, and thus, to contribute to better recognition and intervention for maternal depression.

**Methods:**

In this cross-sectional observational study, baseline data from early pregnancy (< 20 weeks) of the DALI (Vitamin D and Lifestyle Intervention for gestational diabetes mellitus prevention) study were analyzed. Maternal mental health was assessed with the World Health Organization Well-Being Index (WHO–5). Women were classified as having a low (WHO–5 ≤ 50) or high wellbeing.

**Results.:**

A total of 735 pregnant women were included. The prevalence of having a low wellbeing was 27.2%, 95% CI [24.0, 30.4]. Multivariate analysis showed independent associations between low wellbeing and European ethnicity, *OR* = .44, 95% CI [.25, .77], shift work, *OR* = 1.81, 95% CI [1.11, 2.93], insufficient sleep, *OR* = 3.30, 95% CI [1.96, 5.55], self-efficacy, *OR* = .95, 95% CI [.92, .98], social support, *OR* = .94, 95% CI [.90, .99], and pregnancy-related worries (socioeconomic: *OR* = 1.08, 95% CI [1.02, 1.15]; health: *OR* = 1.06, 95% CI [1.01, 1.11]; relationship: *OR* = 1.17, 95% CI [1.05, 1.31]).

**Conclusions:**

Mental health problems are common in European overweight/obese pregnant women. The identified correlates might help in early recognition and subsequent treatment of poor mental health problems during pregnancy. This is important to reduce the unfavorable effects of poor mental health on pregnancy outcomes.

**Trial registration:**

ISRCTN70595832, 02.12.2011.

## Background

The statement of the World Health Organization (WHO) that there can be no “health without mental health” [1] emphasizes the importance of mental health. Almost 30% of all people experience serious mental health problems, such as mood or anxiety disorders, across their lifetime [2]. Depression alone has a cross-cultural lifetime prevalence from 1 to 17% [3] and has a substantial influence both on disability and mortality [4]. It is associated with various adverse health conditions such as unexplained somatic symptoms [5], cardiovascular disease [6, 7], type 2 diabetes [8], HIV/AIDS [9] and tuberculosis [10]. In addition, depression is associated with obesity (body mass index (BMI) ≥ 30 kg/m^2^) [11] and this association becomes stronger at an older age [12].

Moreover, depression is one of the more common complications during pregnancy [13] with a prevalence of up to 11% [14]. Pregnancy-related maternal depression has been found to be associated with obesity [15], poor socioeconomic status (including occupation, education and income), lack of social support, history of domestic violence or abuse, personal history of mental illness, unplanned pregnancy, adverse life events and high perceived stress, smoking, single status, past or present pregnancy complications and pregnancy loss [16, 17]. Furthermore, maternal depression appears to be inversely related with physical activity (PA) [18] and there is evidence to suggest that PA can help to reduce antenatal depression [19] as well as improve maternal physical health [20].

Depression in pregnancy not only affects the health of the woman, but also, potentially, the unborn child. The fetal period is a sensitive and challenging period, where changes and harm could have short- and long-term consequences for the development of the offspring. In contrast to parental genetic factors, fetal programming (e.g., programming of the child’s stress response system) supposes *intra uterine* developmental origins of future health and disease, mainly through epigenetic mechanism [21]. Thus, a stressful in utero environment (e.g., poor maternal mental health) could lead to detrimental consequences for the offspring’s physical and mental health [22, 23].

Indeed, there is substantial evidence that pregnancy-related depression is associated with higher risks for negative child outcomes, potentially lasting until late adolescence [24]. For instance, depression in pregnancy represents a risk factor for adverse birth outcomes such as preterm birth (< 37 weeks of gestation), low birth weight (< 2500 g), small-for-gestational age [25, 26] and poorer behavioral, neurophysiological and cognitive development (e.g., emotional and immune functioning) of the child [27, 28]. Consequently, there is no other period in life where the statement “there is no health without mental health” is more true than during pregnancy [29].

Since obesity and depression are often comorbid [11], it is important to investigate mental health in obese pregnant women. The increasing prevalence of overweight (BMI ≥ 25 kg/m^2^) and obese (BMI ≥ 30 kg/m^2^) pregnant women is by itself already a growing public health concern worldwide [30, 31]. Maternal obesity increases the risk for pregnancy-related complications such as neonatal intensive care requirement, infection and haemorrhage [32] and may even affect future health of the offspring [33]. Although extensive research on the impact of obesity on maternal physical health has been carried out, less attention has been paid to relationship of obesity and maternal mental health. In particular, a better understanding of factors associated with poor mental health in obese pregnant women, and opportunities for early recognition and management is most important.

However, studies concerning mental health in pregnancy were often performed in low-risk populations such as women without serious health conditions and European-wide studies on a broad range of maternal mental health correlates are sadly lacking. This study combines these two aspects with the objective to explore the role of correlates, including lifestyle, socioeconomic, biological, and pregnancy-specific factors, associated with poor mental health in early pregnancy in a large group of overweight/obese European women.

## Methods

### Study design and setting

This cross-sectional observational study was part of the DALI (Vitamin D and Lifestyle Intervention for gestational diabetes mellitus prevention) project (ISRCTN70595832), which aimed to identify the best available preventive intervention (healthy eating (HE), PA, vitamin D) for gestational diabetes mellitus in a European cohort of obese pregnant women. Therefore, following a pilot study [34], a randomized controlled trial was implemented in nine European countries: Austria, Belgium, Denmark, Italy, Ireland, the Netherlands, Poland, Spain and the United Kingdom. Detailed study design and data collection of the DALI project were published previously [35]. Participants were recruited from January 2012 to April 2015 by participating hospitals, obstetrician, midwifes and general practices and randomly allocated to one of the eight intervention arms (HE, PA, HE & PA, HE & PA & vitamin D, HE & PA & placebo, vitamin D alone, placebo alone, control). Measurements took place at baseline (< 20 weeks of gestation), 24–28 weeks, 35–37 weeks and after delivery. Participants receiving the lifestyle interventions were assigned a lifestyle coach and those receiving the vitamin D interventions were asked to take four vitamin D tablets per day (each containing on average 400 IU vitamin D) from randomization until delivery. The lifestyle coaching was based on principles of patient empowerment and cognitive behavioral techniques inspired by motivational interviewing and included five face-to-face sessions, up to four telephone sessions, handbooks and educational materials in order to increase PA, HE or both.

For the purpose of the present study, cross-sectional data from early pregnancy (< 20 weeks; baseline) of the pilot study, Lifestyle trial and Vitamin D trial were used. The study received ethical approval in all nine countries. All participants provided written informed consent.

### Participants

Inclusion criteria were defined as singleton pregnancy, less than 20 weeks of gestation, pre-pregnancy BMI ≥ 29 kg/m^2^ (based on self-reported weight and measured height) or BMI ≥ 29 kg/m^2^ at baseline measurement and age ≥ 18 years. The BMI cut off was based on data on the European obesity prevalence to ensure sufficiency in the recruiting process for countries with lower rates of maternal obesity [36]. Other exclusion criteria were pre-existing diabetes, inability to walk ≥100 m safely, multiple pregnancy, requiring a complex diet, significant chronic medical condition or psychiatric disease, unable to speak the major language of the country of recruitment fluently or unable to converse with the lifestyle coach in another language for which translated materials existed.

Of the 3544 women approached, 1069 (30.2%) consented to participate. Of those, a total of 738 women between 18.7 and 47.1 years (*M* = 32.0, *SD* = 5.4) fulfilled all inclusion criteria and were randomized and included at baseline. Seventy-six (10.3%) were overweight at baseline (BMI < 30). Please note that from now on we refer to the whole sample as *obese*, including this percentage of overweight pregnant women. Three of 738 women did not complete mental health measurements, and therefore, were excluded from the present study. Socio-demographic characteristics are presented in Table 1 in detail. Number of missing data was below 5% for all variables (see Table 1), except for heart rate data (5.6%). Differences between missing and non-missing data were assessed if there were more than 5% missing values for one variable.

**Table 1:** Maternal socio-demographic characteristics

Characteristic	Total (*N* = 735)	Low wellbeing^a^ (*n* = 200)	High wellbeing^a^ (*n* = 535)
Age, *years* (*M* ± *SD*)	32.0 ± 5.4	31.3 ± 5.9	32.3 ± 5.2
BMI, *kg/m* ^*2*^ (median (IQR))	33.4 (31.4–36.6)	33.4 (31.7–36.9)	33.4 (31.3–36.6)
Pre-pregnancy BMI, *kg/m* ^*2*^ (median (IQR))	32.7 (30.5–35.8)	33.0 (30.9–35.7)	32.5 (30.2–35.8)
Ethnicity, *European descent* (*n* (%))	638 (86.8)	160 (80.0)	478 (89.3)
Education (*n* (%), total = 734)			
Low (no qualification/intermediate)	89 (12.1)	28 (14.0)	61 (11.4)
Medium (higher school/apprenticeship)	233 (31.7)	75 (37.5)	158 (29.5)
High (diploma/university)	412 (56.1)	96 (48.0)	316 (59.1)
Occupational status (*n* (%), total = 733)			
Home duties	63 (8.6)	21 (10.5)	42 (7.9)
Unemployed/not able to work	109 (14.8)	37 (18.5)	72 (13.5)
Working (fulltime/part-time/student)	561 (76.3)	141 (70.5)	420 (78.5)
Household composition, *living with partner* (*n* (%))	672 (91.4)	177 (88.5)	495 (92.5)
Marital status, *with partner* (*n* (%), total = 734)	690 (93.9)	179 (89.5)	511 (95.5)
Shift work, *yes* (*n* (%), total = 730)	149 (20.3)	52 (26.0)	97 (18.1)

^a^Based on the World Health Organization Well-Being Index (WHO-5). Values ≤50 were considered as low wellbeing (poor maternal mental health), values >50 as high wellbeing

### Measures

#### Mental health

Assessment of maternal mental health was based on the World Health Organization Well-Being Index (WHO-5), a widely-used, unidimensional instrument measuring subjective wellbeing [37]. It has shown to be a valid screening instrument for clinical depression with high sensitivity and specificity, and has been validated for all languages required in the present multi-national study [38, 39].The scale consists of five items, each rated on a 6-point Likert scale (0: at no time, 5: all of the time), and following total scores, standardized scores (0–100) are calculated. Based on current literature on sensitivity and specificity for clinical depression, values ≤50 were considered as low wellbeing (poor maternal mental health), values above 50 as high wellbeing [38]. Cronbach Alpha (α) was 0.82 for the whole scale.

### Potential correlates

The selection of variables collected at baseline was based on current literature [7, 16, 17] and then clustered into 4 groups: a) socio-demographic (age, pre-pregnancy BMI, BMI at baseline, ethnicity, education, household composition, occupational status, shift work, marital status), b) lifestyle (smoking, alcohol consumption, hours of sleep per day (24 h), days of insufficient sleep per month, snoring, PA), c) biological (polycystic ovary syndrome, first degree relative with diabetes, fasting plasma glucose, fasting plasma insulin, Homeostasis Model Assessment of Insulin Resistance (HOMA-IR), systolic and diastolic blood pressure, chronic hypertension, resting heart rate) and d) pregnancy characteristics (weeks of gestation (at baseline), pregnant before, number of own children, previous stillbirths/miscarriages, previous fetal macrosomia, previous congenital malformation, previous gestational diabetes, pre-pregnancy to baseline weight gain, attitude/importance of current weight, social support, outcome expectancies, self-efficacy, pregnancy-related worries). Physiological parameters (plasma glucose, plasma insulin, blood pressure, heart rate) were quantified by standardized, commercially available devices, whereas all other variables were self-reported.

Hours of sleep per day (24 h), snoring and insufficient sleep were retrieved from single items (How many hours do you sleep per day on average; How many days in the last month have you had the feeling of insufficient sleep; How many days per week do you snore/are told you snore). Sleep hours per day were categorized into ≤6 h, 6–9 h and ≥9 h [40], ethnicity into European and non-European, education into low (no qualification/intermediate), medium (higher school/apprenticeship) and high (diploma/university), occupational status into working, not working and home duties, household composition into living with partner and not living with partner, marital status into with partner and without partner.

PA was based on the pregnancy physical activity questionnaire (PPAQ), which is a valid instrument for the assessment of PA during pregnancy [41]. In addition to 32 original activities, two more questions concerning *cycling to work* and *cycling for fun* have been added. Time spent in each activity was asked, and then multiplied by its intensity, resulting in an average weekly energy expenditure (MET hours/week) for each activity. After categorization of activities into sedentary, light, moderate or vigorous, total MET hours/week of moderate-to-vigorous physical activity (MVPA) and sedentary behavior (SB) were used for analysis.

Pregnancy-related worries have shown to be related to mood outcomes and were assessed by the Cambridge Worry Scale [42], a 13-item questionnaire (6-point Likert scale; 0: not a worry, 5: major worry) measuring worries across four domains: sociomedical (α = .71), socioeconomic (α = .59), health (α = .66) and relationship (α = .61). Cronbach Alpha for the whole scale was .77. Domain scores were used for analysis. Attitude (e.g., *It is important for me to manage my weight*), social support (e.g., *I am satisfied with the level of support I am receiving for eating healthily from my partner, family and friends*), outcome expectancies (e.g., *Staying physically active during this pregnancy, will help to reduce health risks for my baby*) and self-efficacy (e.g., *I am confident that I will succeed in managing my weight*) were based on the Health Action Process Approach (HAPA) model [43], referring to PA, nutrition and weight management during pregnancy, and collected with items on a 10-point Likert scale. Attitude to current weight as well as social support consisted of two items (α_a_ = .76, α_s_ = .87), outcome expectancies of six items (α = .93) and self-efficacy of five items (α = .88). Sum scores of each dimension were used for further analysis.

### Statistical analysis

Statistical analyses were performed using SPSS Data Analysis version 22.0 (IBM Corp, Armonk, NY, USA). Descriptive statistics for all variables were calculated, including mean and standard deviation for continuous variables with normal distribution, median and interquartile range (IQR) for continuous variables without normal distribution, and frequencies and percentages for categorical variables. Normal distribution was assessed by Shapiro-Wilk, Q-Q-Plots and Histogram. Evaluation of missing and non-missing data was done by *t* test, Welch test and Chi square or Fisher’s exact tests.

To evaluate the association between poor maternal mental health and potential correlates, logistic regression models were used, comprising a three-step procedure. In all analysis, the outcome was defined as the dichotomous variable derived from the WHO-5 index (low wellbeing vs. high wellbeing). Odds ratios (*OR*) with 95% confidence intervals (CI) were calculated for each association. In a first step, bivariate logistic regression models between outcome and each potential correlate were calculated. Significant correlates were included in the next step. In this second step, four multivariate logistic regression models were calculated, set up of blocks with related correlates (1) socioeconomic status (2) sleep (3) worries (4) perceptions and attitudes. All correlates of one block were entered at the same time and significant correlates were included in the next step. In this third and final step, remaining correlates were analyzed in one multivariate logistic regression model, entering all correlates at the same time.

Significance in step one and two was considered as *p* < .10 to allay beta error. In step 3 as well as in all other analyses, significance was considered as *p* < .05. BMI at baseline (based on measured weight and height) and pre-pregnancy BMI (based on self-reported weight and measured height) were both included in the first step, but if significant, BMI at baseline was used for step two and three. Linearity between outcome and each potential correlate was assessed by the interaction term between the potential correlate and its log transformation within the binary analysis, and if significant, tertiles of the predictor were calculated. To consider the possible clustering effect within countries, multilevel analysis with a random intercept was performed with Stata 12 (StataCorp, College Station, TX, USA). Multilevel analysis was carried out for the final model (step three), including a null model (random-intercept-only) and a random intercept model.

## Results

### Sample characteristics

Two hundred women (27.2%, 95% CI [24.0, 30.4]) had a “low wellbeing” and 535 women (72.8%) had a “high wellbeing” based on the pre-defined WHO-5 scores. In total, 561 women (76.5%) were working and 690 (94.0%) had a partner. 412 (56.1%) women were highly educated (diploma or university), whereas 89 (12.1%) had only a low education (no qualification or intermediate). Sample characteristics are displayed in Table 1 and Table 2 in detail.

**Table 2:** Specific maternal characteristics

Characteristic	Total (*N* = 735)	Low wellbeing^a^ (*n* = 200)	High wellbeing^a^ (*n* = 535)
Lifestyle			
Smoking behaviour, *yes* (*n* (%))	122 (16.6)	37 (18.5)	85 (15.9)
Any alcohol consumption, *yes* (*n* (%))	43 (5.9)	12 (6.0)	31 (5.8)
Sleep, *hours per day* (median (IQR), total = 734)	8.0 (7.0–8.5)	8.0 (7.0–9.0)	8.0 (7.0–8.0)
Insufficient sleep, *days per month* (median (IQR, total = 722)	7.0 (3.0–15.0)	15.0 (5.0–20.0)	5.0 (2.0–15.0)
Snoring, *days per week* (median (IQR, total = 714)	0.0 (0–2.0)	0.0 (0–3.0)	0.0 (0–2.0)
PPAQ, *MET-h·wk.* ^*−1*^ (median (IQR))			
MVPA	70.2 (34.7–127.8)	74.7 (32.3–128.2)	69.0 (35.5–127.8)
SB	42.0 (19.1–62.1)	36.8 (18.6–61.1)	43.2 (19.4–62.7)
Biological			
First degree relative with diabetes, *yes* (*n* (%))	168 (22.9)	48 (24.0)	120 (22.4)
Chronic hypertension, *yes* (*n* (%), total = 730)	98 (13.3)	30 (15.0)	68 (12.7)
Polycystic ovary syndrome, *yes* (*n* (%), total = 728)	73 (9.9)	24 (12.0)	49 (9.2)
Fasting plasma glucose, *mmol/L (*median (IQR), total = 727)	4.6 (4.3–4.8)	4.6 (4.3–4.8)	4.6 (4.3–4.8)
Fasting plasma insulin, *mmol/L (*median (IQR), total = 720)	12.7 (9.8–17.5)	13.1 (10.1–17.5)	12.5 (9.5–17.4)
HOMA-IR (median (IQR), total = 717)	2.6 (1.9–3.6)	2.7 (2.0–3.6)	2.6 (1.9–3.6)
Systolic blood pressure (median (IQR), total = 733)	116 (109–123)	114 (107–121)	116 (110–124)
Diastolic blood pressure (median (IQR), total = 733)	72 (67–79)	71 (67–77)	72 (67–79)
Resting heart rate (median (IQR), total = 694)	79 (72–86)	79 (71–87)	79 (73–85)
Pregnancy-specific			
Gestational week (median (IQR))	15.1 (13.4–16.7)	14.9 (13.3–16.2)	15.3 (13.6–17.0)
Number of own children (*n* (%), total = 734)			
Zero	366 (49.8)	93 (46.5)	273 (51.0)
One	256 (34.8)	73 (36.5)	183 (34.2)
Two or more	112 (15.2)	34 (17.0)	78 (14.6)
Pregnant before, *yes* (*n* (%))	460 (62.6)	136 (68.0)	324 (60.6)
Previous stillbirths/miscarriage, *≥ 1* (*n* (%), total = 445)	200 (27.2)	63 (31.5)	137 (25.6)
Previous fetal macrosomia, *yes* (*n* (%), total = 453)	81 (11.0)	22 (11.0)	59 (11.0)
Previous congenital malformation, *yes* (*n* (%), total = 454)	16 (2.2)	4 (2.0)	12 (2.2)
Previous gestational diabetes, yes (*n* (%), total = 455)	36 (4.9)	12 (6.0)	24 (4.5)
Pre to baseline weight gain, *kg* (median (IQR))	1.7 (−0.4–4.0)	1.6 (−0.6–4.2)	1.7 (−0.3–4.0)
Perceptions and attitude^b^ (median (IQR))			
Attitude to current weight (total = 729)	16.0 (14.0–18.0)	17.0 (14.0–19.0)	16.0 (13.0–18.0)
Self-efficacy (total = 730)	35.0 (30.0–41.0)	31.0 (27.0–38.0)	37.0 (31.0–41.0)
Social support (total = 731)	16.0 (13.0–18.0)	14.0 (10.0–17.0)	16.0 (14.0–18.0)
Outcome expectancy (total = 723)	52.0 (46.0–58.0)	52.0 (46.0–58.0)	52.0 (46.0–58.0)
Cambridge worry scale (median (IQR))			
Total 13 items (total = 720)	18.0 (12.0–26.0)	24.0 (16.0–32.0)	17.0 (12.0–24.0)
Sociomedical (total = 731)	5.0 (2.0–8.0)	6.0 (2.0–10.0)	5.0 (2.0–8.0)
Socioeconomic (total = 733)	4.0 (2.0–7.0)	6.0 (3.0–9.0)	4.0 (1.0–6.0)
Health (total = 729)	8.0 (5.0–12.0)	10.0 (6.0–13.0)	8.0 (5.0–11.0)
Relationship (total = 729)	0.0 (0–2.0)	1.0 (0–3.0)	0.0 (0–1.0)

PPAQ = pregnancy physical activity questionnaire; MET = metabolic equivalents; MVPA = moderate-to-vigorous physical activity; SB = sedentary behavior

^a^Based on the World Health Organization Well-Being Index (WHO-5). Values ≤50 were considered as low wellbeing (poor maternal mental health), values >50 as high wellbeing. ^b^Based on the Health Action Process Approach (HAPA) model [43]

Comparison of missing and non-missing data of resting heart rate showed no significant differences (*p* ≥ .05) in age, education, ethnicity, household composition, shift work, marital status, BMI, gestational week, pregnancy-related worries, sleep hours, systolic and diastolic blood pressure. Nonetheless, significant differences were found between missing/non-missing data of resting heart rate and maternal mental health (*p* < .01) and occupational status (*p* < .05).

### Bivariate analysis

Bivariate logistic regression models (first step) showed significant associations (*p* < .10, data not shown) between low wellbeing and age, BMI at baseline, weeks of gestation, ethnicity, household composition, education, occupational status, attitude to current weight, self-efficacy, social support, pregnancy-related worries (sociomedical, socioeconomic, health, relationship), marital status, pregnant before, shift work, hours of sleep per day, days of insufficient sleep per month, snoring, and systolic blood pressure.

No significant associations (*p* ≥ .10, data not shown) were displayed between low wellbeing and smoking, alcohol consumption, outcome expectancies, number of own children, previous stillbirths/miscarriages, previous fetal macrosomia, previous congenital malformation, PA, previous gestational diabetes, chronic hypertension, polycystic ovary syndrome, pre to baseline weight gain, fasting plasma glucose, fasting plasma insulin, HOMA-IR, diastolic blood pressure, and resting heart rate.

### Multivariate analysis

In a second step, four multivariate models (a-d) were developed. Remaining variables associated with socioeconomic status (ethnicity, household composition, education, occupational status, marital status, shift work) were analyzed in a first model (a). This model, χ^2^ (8, *N* = 727) = 29.79, *p* < .01, *R* ^*2*^ = .04, showed significant associations between low wellbeing and ethnicity (European vs. not European: *OR* = .52, 95% CI [.33, .83], *p* < .01), occupational status (not working vs. working: *OR* = 1.51, 95% CI [.94, 2.41], *p* = .09), marital status (with vs. without partner: *OR* = .39, 95% CI [.18, .88], *p* = .02) and shift work (yes vs. no: *OR* = 1.79, 95% CI [1.19, 2.69], *p* < .01), but no longer with household composition (*p* = .53) and education (low: *p* = .61, medium: *p* = .13).

The second model (b), χ^2^ (5, *N* = 704) = 53.44, *p* < .01, *R* ^*2*^ = .07, consisting of remaining variables related to sleeping quality (hours of sleep, days of insufficient sleep, snoring), showed significant associations of low wellbeing with hours of sleep per day (9 or more vs. normal: *OR* = 1.60, 95% CI [1.05, 2.45], *p* = .03) and days of insufficient sleep per month (moderate vs. low: *OR* = 1.73, 95% CI [1.08, 2.77], *p* = .02; high vs. low: *OR* = 4.13, 95% CI [2.63, 6.48], *p* < .01), but no longer with snoring (*p* = .32).

The third model (c), χ^2^ (4, *N* = 720) = 72.94, *p* < .01, *R* ^*2*^ = .10, referring to remaining variables of the Cambridge Worry Scale, showed significant associations of low wellbeing with socioeconomic worries (*OR* = 1.11, 95% CI [1.05, 1.17], *p* < .01), health-related worries (*OR* = 1.06, 95% CI [1.02, 1.11], *p* < .01) and relationship-related worries (*OR* = 1.24, 95% CI [1.12, 1.37], *p* < .01), but no longer with sociomedical worries (*p* = .90).

The fourth model (d), χ^2^ (3, *N* = 725) = 66.49, *p* < .01, *R* ^*2*^ = .09, referring to variables from the HAPA model, showed significant associations of low wellbeing and attitude to current weight (*OR* = 1.10, 95% CI [1.05, 1.16], *p* < .01), self-efficacy (*OR* = .96, 95% CI [.93, .98], *p* < .01) as well as social support (*OR* = .91, 95% CI [.87, .95], *p* < .01).

In the third and final step, all remaining variables of step two were analyzed within one multivariate logistic regression model. Results are displayed in Table 3, showing that low wellbeing was significantly associated with non-European ethnicity, shift work, insufficient sleep, low self-efficacy, low social support and pregnancy-related worries (socioeconomic, health, relationship), χ^2^ (21, *N* = 692) = 163.71, *p* < .001, *R* ^2^ = .21.

**Table 3:** Multivariate logistic regression. Associations of low wellbeing and maternal characteristics

	Odds Ratio, 95% CI			
*Maternal characteristic*	*OR*	Lower	Upper	*p*
Ethnicity (European)	.44	.25	.77	< .01*
Marital status (with partner)	.45	.20	1.02	.06
Shift work (yes)	1.81	1.11	2.93	.02*
Occupational status (working = ref)				
Not working	1.39	.80	2.42	.24
Home duties	1.28	.59	2.78	.53
Sleep h/d (6–9 h = ref)^a^				
≤ 6 h	.90	.52	1.56	.70
≥ 9 h	1.11	.68	1.84	.67
Insufficient sleep d/m (low = ref)^b^				
Medium	1.26	.73	2.17	.41
High	3.30	1.96	5.55	< .01*
Perceptions and attitude^c^				
Attitude to current weight	1.05	.99	1.12	.11
Self-efficacy	.95	.92	.98	< .01*
Social support	.94	.90	.99	.03*
Cambridge worry scale				
Socioeconomic	1.08	1.02	1.15	.01*
Health	1.06	1.01	1.11	.03*
Relationship	1.17	1.05	1.31	< .01*
Age (young = ref)^d^				
middle	.81	.50	1.31	.39
old	.90	.54	1.49	.68
BMI at baseline	1.03	.98	1.08	.32
Weeks of gestation	.95	.87	1.03	.17
Pregnant before (yes)	1.11	.72	1.71	.64
Systolic blood pressure	.99	.97	1.01	.21

Only variables remaining after step two were included in the model (method = enter, outcome = WHO-5 index: low wellbeing), χ2 (21, *N* = 692) = 163.71, *p* < .001, *R* ^2^ = .21, Durbin Watson = 2.10

^a^sleep hours per day (24 h, self-reported); ≤ 6 h; 6–9 h; ≥ 9 h

^b^days per month (self-reported), based on tertiles

^c^Based on the Health Action Process Approach (HAPA) model [43]

^d^based on tertiles

*< .05

Although multilevel analysis revealed significant level 2 variances for country, τ_00_ = .10, 95% CI [.02, .48], ρ = .03, *p* < .05, in the null model (random-intercept-only), there was no significant improvement of the final model in step 3, when adding a random intercept. Therefore, results of step 3 are presented without a random intercept (Table 3).

## Discussion

In summary, this study showed independent associations between low wellbeing and non-European ethnicity, shift work, insufficient sleep, low self-efficacy, low social support and pregnancy-related worries (socioeconomic, health, relationship) in obese pregnant women.

A recent meta-analysis [15] on the prevalence of depressive symptoms in pregnancy showed higher rates for obese (33%) than normal-weight pregnant women (22.6%). Likewise, in the present study, 27.2% of all women with BMI ≥ 29 had a low wellbeing. There is a reciprocal relationship between poor mental health and obesity [11] and both have been linked with numerous adverse pregnancy outcomes such as neonatal intensive care requirement, preterm birth and gestational diabetes [24, 32, 33]. Thus, healthcare providers should be aware of the high prevalence of obese pregnant women experiencing mental health problems.

A number of studies reported a broad range of potential correlates of maternal mental health [16, 17], although some variability appears, largely based either on bivariate or multivariate analysis, sample composition and study design. For example, the present study demonstrated an association between maternal mental health and European ethnicity, which is in line with previous studies, showing both independent and mutual influences of ethnicity and variables of socioeconomic status (e.g., occupation, education, income) on maternal mental health [16, 44, 45]. Likewise, sleep quality is associated with depression in the general population [46] and during pregnancy, where poor sleep quality and sleep loss are linked with a greater risk of both maternal mood problems [47] and adverse birth outcomes [48]. This confirms our finding of the linkage between low wellbeing and insufficient sleep. In addition, previous studies confirm our findings of associations between low wellbeing and individual variables such as low self-efficacy, worrying and less perceived social support as well [16].

In contrast to other studies, we did not find associations (neither univariate nor multivariate) for previously described correlates of mental health in pregnancy such as age, alcohol consumption, smoking, number of own children or previous pregnancy complications [16, 17]. This may be due to the selection of women participating in the DALI study, small frequencies for some correlates (e.g., alcohol consumption, previous congenital malformation, previous gestational diabetes) or restriction of inclusion for BMI and gestational week.

Interestingly, no association between low wellbeing and PA was found, in contrast to previous research [18, 49, 50]. This may be related to the study context, where women were included in a lifestyle trial, partly focused on physically activity. Moreover, we studied women in early pregnancy, while the decrease in PA may be more pronounced later in pregnancy [18].

According to pregnancy-related worries, pregnancy is a sensitive period in a woman’s life, and is likely to evoke heightened levels of anxiety and worrying [51] with unique influences on mood outcomes [42]. As shown in the present study, pregnancy-related worries should be considered as possible correlates of poor maternal mental health states. Furthermore, it may be possible that pregnancy-related worries play a crucial role in intervention strategies on improving maternal mental health. However, in pursuance of achieving the best outcomes when developing such intervention strategies, more insight into the relevance of a woman’s current worries in pregnancy and how to deal with them is needed.

Under the terms of the health action process approach [43], social support and self-efficacy are important factors for forming intentions and maintaining changes in health behaviors (e.g., for PA or HE). Since many studies reported an inverse relationship between depression in pregnancy and PA [18], it could be possible that lack of social support and low self-efficacy deteriorate the unfavorable effects of an unhealthy lifestyle on mental and physical health. These effects might be even stronger in late pregnancy. Considering low social support and self-efficacy as correlates might help for an early recognition of poor maternal mental health states and the design of interventions. In particular, implementing intervention strategies, which include for instance, participation of family members and friends in order to increase social support or specific tasks to increase perceived self-efficacy are advisable. The inclusion of such individual characteristics into tailored intervention strategies would probably result in the best outcomes of maternal mental health.

Finally, the well-known association between poor mental health and shift work [52] was confirmed in pregnancy as well. In short, working in shifts (e.g., night work, rotating shifts) disturbs the circadian rhythm, a dynamic system which regulates most of our physiological processes based on the day-night cycle, and which is associated with pregnancy complications such as pre-term birth or low birth weight [53]. Together with sleep deprivation, the disruption of the circadian system could act as stressor with detrimental consequences on many body systems, including woman’s reproductive function, while leading to a cumulative wear out of the whole system [54]. With this in view, integrating shift work as a correlate of poor maternal mental health is highly recommended.

During the last decade, researchers have shown an increased interest in mental health during pregnancy. However, the use of maternal mental health correlates for early recognition of poor mental health into clinical practice seems to be limited as yet. Clearly, some of the identified correlates would be difficult to routinely assess in clinical practice but others (e.g., non-European ethnicity, worrying, insufficient sleep, shift work) could be easily assessed without the requirement of laboratory equipment or standardized questionnaires. Such an implementation (e.g., for screening) would be possible both in primary (e.g., general practitioners, midwifes) and secondary health care settings (obstetricians) and could help in early recognition of maternal mental health problems at a low threshold. In addition, identified correlates give direction to further intervention development for prevention or treatment of mental health problems in pregnancy. Finally, because of the reciprocal relationship between obesity and poor mental health, tackling mental health problems of obese pregnant women might help to mitigate part of the adverse effects of obesity on pregnancy outcomes.

### Limitations and strengths

Our study has limitations and strengths that deserve to be mentioned. First, due to the cross-sectional design, no conclusions about causality can be drawn. To confirm whether the identified factors are indeed predictors for low mood in the course of pregnancy and to establish the consequences for both the woman and the child, longitudinal studies are warranted. In addition, the cross-sectional design and the chosen analysis strategy hinders the detection of causal pathways of variables associated with mental health in pregnancy. However, this study focused on the detection of correlates of poor mental health rather than causal pathways. Secondly, our data were derived from a large group of women from different European countries participating in the DALI study who might have a better mental health compared to others, possibly leading to a selection bias.

Yet, 27% reported low wellbeing, as defined by their WHO-5 score, which is in concert with previous research on other populations, and thus does not suggest an underestimation. Of course, we need to acknowledge that we used a relatively short measure of mental health (subjective wellbeing) rather than a diagnostic interview, which is the gold standard for establishing the true prevalence of major depression. Such a procedure was not feasible in the context of this large multi-national lifestyle study. Regardless, the WHO-5 scale has shown good screening properties for clinically relevant depressive symptoms, was used worldwide and tested in all languages required in the present study, as well as variations of the scale dependent on country of recruitment were statistically evaluated in the present study [38, 39]. Therefore, a bias is very unlikely and our data can be regarded as valid. Future studies should aim to thoroughly investigate the prevalence and course of minor and major depression in pregnancy.

Nonetheless, to our knowledge, this is the first study, which investigated a broad range of potential correlates of poor maternal mental health in a crucial period such as the early pregnancy in conjunction with a wide and representative European sample.

## Conclusions

One of the great challenges is to recognize maternal mental health problems very early in pregnancy in order to prevent harmful health consequences. This study showed that mental health problems are common in European obese pregnant women (27.2%) and that non-European ethnicity, having shift work, insufficient sleep, low self-efficacy, low social support, and pregnancy-related worries have to be considered as correlates of poor maternal mental health in this population. Other factors such as smoking, alcohol consumption, marital status, occupational status and maternal age do not seem be associated with maternal mental health in this population. Some of these identified correlates may further help for an early recognition in clinical practice and the design of interventions. Especially insufficient sleep and non-European ethnicity seem to play a prominent role according to their effect sizes.

## Abbreviations

BMI: body mass index
HAPA: health action process approach
HE: healthy eating
HOMA-IR: homeostasis model assessment of insulin resistance
IQR: interquartile range
MET: metabolic equivalent of task
MVPA: moderate-to-vigorous physical activity
PA: physical activity
PPAQ: pregnancy physical activity questionnaire
SB: sedentary behavior
WHO: world health organization
